# Thirty Days of Montmorency Tart Cherry Supplementation Has No Effect on Gut Microbiome Composition, Inflammation, or Glycemic Control in Healthy Adults

**DOI:** 10.3389/fnut.2021.733057

**Published:** 2021-09-16

**Authors:** Angela R. Hillman, Bryna C. R. Chrismas

**Affiliations:** ^1^School of Applied Health Sciences and Wellness, Division of Exercise Physiology, Ohio University, Athens, OH, United States; ^2^College of Education, Department of Physical Education, Qatar University, Doha, Qatar

**Keywords:** polyphenol, cherry, montmorency, microbiome, glucose, inflammation

## Abstract

Tart cherries possess properties that may reduce inflammation and improve glycemic control, however human data on supplementation and the gut microbiota is equivocal. Processing (i.e., juice concentrate, dried, frozen) may affect the properties of tart cherries, and therefore alter their efficacious health benefits. Therefore, the purpose of this study was to investigate the effect of 30 days of supplementation with Montmorency tart cherry (MTC) in concentrate or freeze-dried form on the gut microbiome and markers of inflammation and glycemic control. Healthy participants with no known disease (*n* = 58, age: 28 ± 10 y, height: 169.76 ± 8.55 cm, body mass: 72.2 ± 12.9 kg) were randomly allocated to four groups and consumed either concentrate or freeze-dried capsules or their corresponding placebos for 30 days. Venous blood samples were drawn at baseline, day 7, 14, and 30 and analyzed for inflammatory markers TNF-alpha, uric acid, C-reactive protein, and erythrocyte sedimentation rate and glycemic control markers glycated albumin, glucose and insulin. A fecal sample was provided at baseline, day 14 and 30 for microbiome analysis. TNF-alpha was significantly lower at 30 vs. 14 days (*p* = 0.01), however there was no other significant change in the inflammatory markers. Insulin was not changed over time (*p* = 0.16) or between groups (*p* = 0.24), nor was glycated albumin different over time (*p* = 0.08) or between groups (*p* = 0.56), however glucose levels increased (*p* < 0.001) from baseline (4.79 ± 1.00 mmol·L^−1^) to 14 days (5.21 ± 1.02 mmol·L^−1^) and 30 days (5.61 ± 1.22 mmol·L^−1^) but this was no different between groups (*p* = 0.33). There was no significant change in composition of bacterial phyla, families, or subfamilies for the duration of this study nor was there a change in species richness. These data suggest that 30 days of MTC supplementation does not modulate the gut microbiome, inflammation, or improve glycemic control in a healthy, diverse group of adults.

**Clinical Trail Registration:**https://clinicaltrials.gov/ct2/show/NCT04467372, identifier: NCT04467372.

## Introduction

A considerable amount of research has focused on the use of foods and supplements containing anthocyanins and polyphenols to reduce disease risk by mitigating inflammation and improving blood glucose regulation. Globally the incidence of type 2 diabetes is rising and is predicted to rise to nearly 700 million by 2,045 (1). Evidence suggests a diet high in polyphenols and or supplementation with polyphenol-containing supplements can improve risk of developing type 2 diabetes (2), which may be in part be due to the gut microbiota (3). Polyphenols are often found in fruits, spices and herbs, vegetables, and drinks. The microbiota are known to metabolize many polyphenols, altering their bioavailability (4), providing energy and metabolites to surrounding enterocytes, and decreasing systemic inflammation (5). While the gut microbiota of adults is typically constant, changes have been observed from dietary interventions (6), including with polyphenol supplementation. A variety of polyphenol containing substances have been investigated for their role in modulating the microbiota, including cocoa, red wine, powdered blueberries, grape seed extract, and tart cherries, with mixed findings. For example, 4 weeks of supplementing with a high-cocoa flavanol supplement increased bifidobacterial and lactobacilli population, while also decreasing C-reactive protein levels, which was primarily driven by the increased lactobacilli counts (7). Similar findings have been found with red wine consumption for 4 weeks, with increases in bifidobacterial counts driving a decrease in C-reactive protein (8). These authors also found red wine ingestion increased *Bacteroides* counts. Yamakoshi et al. (9) found 14 days of supplementing with proanthocyanidin-rich grape seed extract increased *Bifidobacterium* bacterial counts, findings corroborated by Vendrame (10) after 6 weeks of powdered wild blueberry supplementation. This study is particularly interesting, because the authors concluded that the added fiber and high polyphenol content of the powdered supplement led to selective increases in *Bifidobacterium* (10), which may also occur with freeze-dried powdered Montmorency tart cherry (MTC) supplementation. However, a more recent study investigating MTC concentrate supplementation for 30 days failed to find any changes in bacteria counts. These authors also noted very low levels of *Bifidobacterium* and *Lactobacilli* spp. at baseline, which may have led to the lack of significant change. However, Mayta-Apaza found only 5 days of supplementing with MTC concentrate increased *Bacteroidetes* and decreased *Firmicutes* in those who had low *Bacteroidetes* counts at baseline (11). The significance of these changes lies in the fact that Bifidobacteria and Lactobacillus are both known to be probiotics, with a plethora of health benefits (12), while many species of Bacteroidetes are important for digestion and a decrease in their relative abundance along with increases in Firmicutes has been seen in obesity (13). Clearly there are equivocal findings in the literature related to gut microbiome changes with MTC that need further exploration given the potential health impacts. In addition, results of polyphenol supplementation studies are equivocal and assessing different formulations is warranted.

MTC concentrate and freeze-dried powder are known to have high levels of proanthocyanidins, phenolics and antioxidant capacity (14). Unfortunately, there has been no comparison between formulations of tart cherry supplements (freeze-dried powder, concentrate, juice, whole) on inflammation and the gut microbiome *in vivo*. This is particularly important in the realm of inflammation research because sugar is known to increase the inflammatory response (15, 16) and the freeze-dried powder form of MTC supplements is naturally low in sugar (<1 g) while the juice (variable, depending on formulation) and concentrate (15+ g) are considerably higher. Furthermore, the addition of the skins in freeze-dried cherry powder supplements would increase fiber content and provide polysaccharides as an energy source to *Bacteroides* (17). While studies of tart cherry have been equivocal on changes in markers of inflammation with variable findings for changes in uric acid (18–23), and C-reactive protein (19–22, 24, 25), investigating the role the gut microbiome may have in modulating the inflammatory response is warranted. In terms of glucose regulation, MTC extract treatment has been shown to inhibit key enzymes in carbohydrate digestion activity, while increasing translocation of glucose transporters, thus improving insulin sensitivity in an *in-vitro* model (26). However, the published human investigations of MTC and glucose regulation in healthy populations have been equivocal. For example, Lear (25) found no change in fasting glucose or insulin, but the Matsuda index, a marker of insulin sensitivity, decreased after 30 days of supplementation, while Desai (27) found no change in glucose and Chai (28) actually found an increase in glucose and decrease in insulin levels after MTC supplementation. Given these equivocal findings on changes in inflammation and glucose regulation with MTC supplementation, further investigation is warranted.

Therefore, the purpose of this study was to examine the effects of 30 days of supplementing with MTC concentrate or freeze-dried powder on the gut microbiome, inflammation, and glucose regulation. We hypothesized that the polyphenols in the MTC products would influence the gut microbiome composition, which would modulate changes in inflammatory markers and glucose regulation.

## Materials and Methods

### Participants

**Figure 1:**
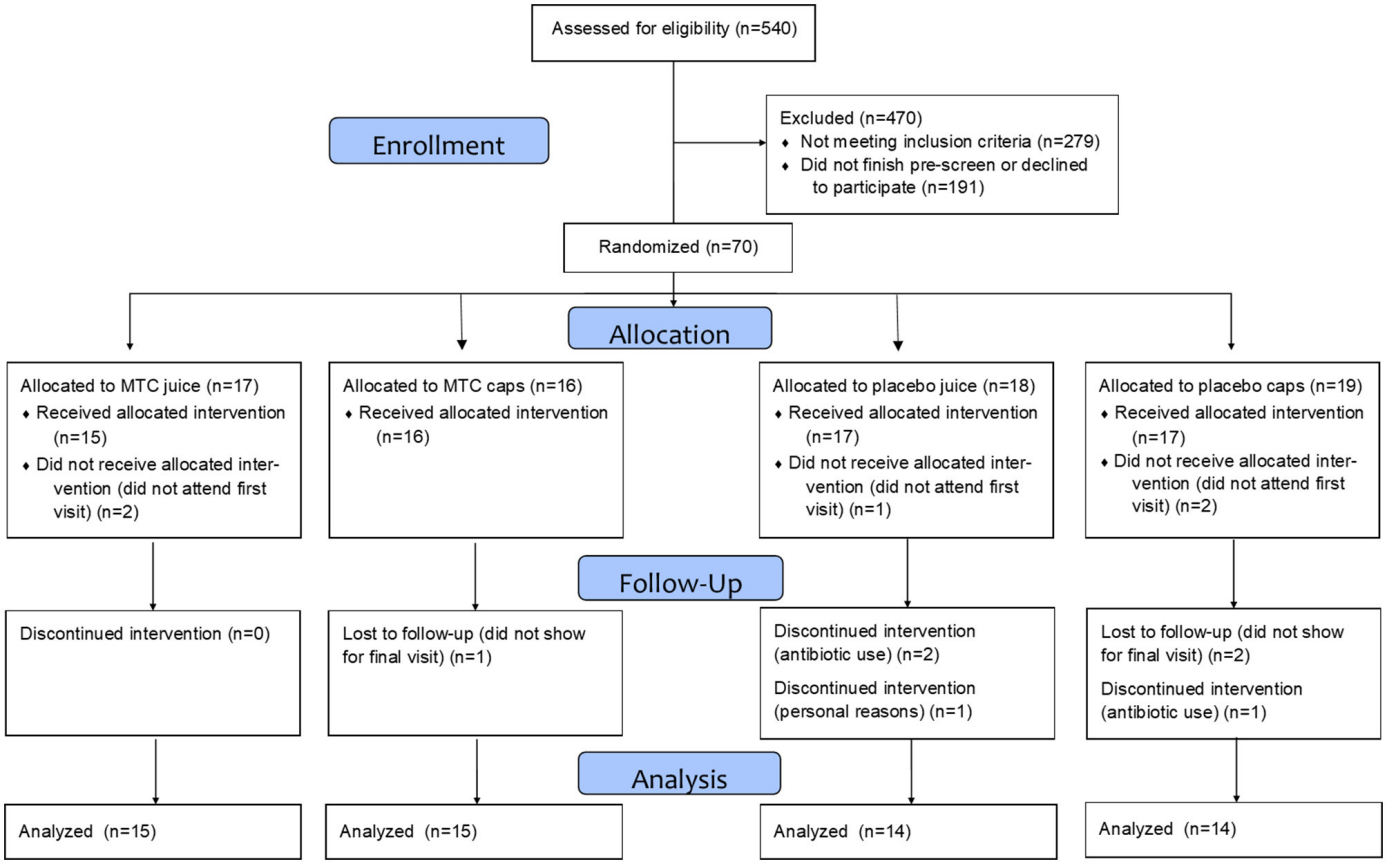
CONSORT diagram for the study.

This study was approved by the Institutional Review Board at Ohio University (IRB# 18-F-13) and written informed consent was obtained from each respondent prior to entering the study. Participants were recruited with a University-wide email and flyers hung on campus and in the community, then pre-screened for inclusion/exclusion criteria *via* an online survey and eligibility was confirmed at the baseline visit. Inclusion criteria included being aged 18–50 years, not pregnant, not diabetic, with no unresolved infections or diseases (diabetes, cardiovascular disease, inflammatory or autoimmune disease), and non-smokers. Participants were also free from prescribed anti-inflammatory and corticosteroid use for at least 2 months and had not taken antibiotics within the last year. See Figure 1 for the CONSORT diagram of recruitment and retention and Table 1 for participant demographics.

**Table 1:** Participant demographics.

	**Juice**		**Capsule**	
	**Placebo**	**MTC**	**Placebo**	**MTC**
Males, females	8, 6	6, 9	4, 10	5, 10
Age (years)	26 ± 7	26 ± 7	27 ± 12	33 ± 13
Height (cm)	171 ± 10	172 ± 9	168 ± 7	169 ± 7
Weight (kg)	74 ± 12	72 ± 15	73 ± 12	70 ± 13
Body fat (%)	22 ± 10	22 ± 6	30 ± 10	24 ± 8
BMI (kg·m^2^)	25 ± 3	24 ± 3	25 ± 4	24 ± 3
Systolic BP (mmHg)	122 ± 10	120 ± 10	121 ± 15	116 ± 10
Diastolic BP (mmHg)	72 ± 9	68 ± 7	72 ± 9	72 ± 8
Fasting blood glucose (mg·dL^−1^)	79 ± 18	88 ± 16	91 ± 17	99 ± 20
Baseline GA (%)	7.7 ± 3.6	7.6 ± 6.2	6.8 ± 5.2	6.0 ± 4.4
Baseline ESR (mm/h)	7 ± 6	7 ± 5	7 ± 6	7 ± 5
Baseline CRP (mg·dL^−1^)	0.2 ± 0.3	0.3 ± 0.5	0.2 ± 0.4	0.2 ± 0.3
Baseline UA (mg·dL^−1^)	5.4 ± 1.3	4.5 ± 1.2	5.0 ± 0.7	5.0 ± 1.3
Average METmin activity/week	2,266 ± 1,565	2,700 ± 1,525	2,363 ± 1,166	3,323 ± 1,501

*MTC, Montmorency tart cherry; BMI, Body mass index; BP, Blood pressure; GA, Glycated albumin; ESR, Erythrocyte sedimentation rate; CRP, C-reactive protein; UA, Uric Acid; METmin, Metabolic equivalent minutes of exercise*.

### Experimental Procedures

**Figure 2:**
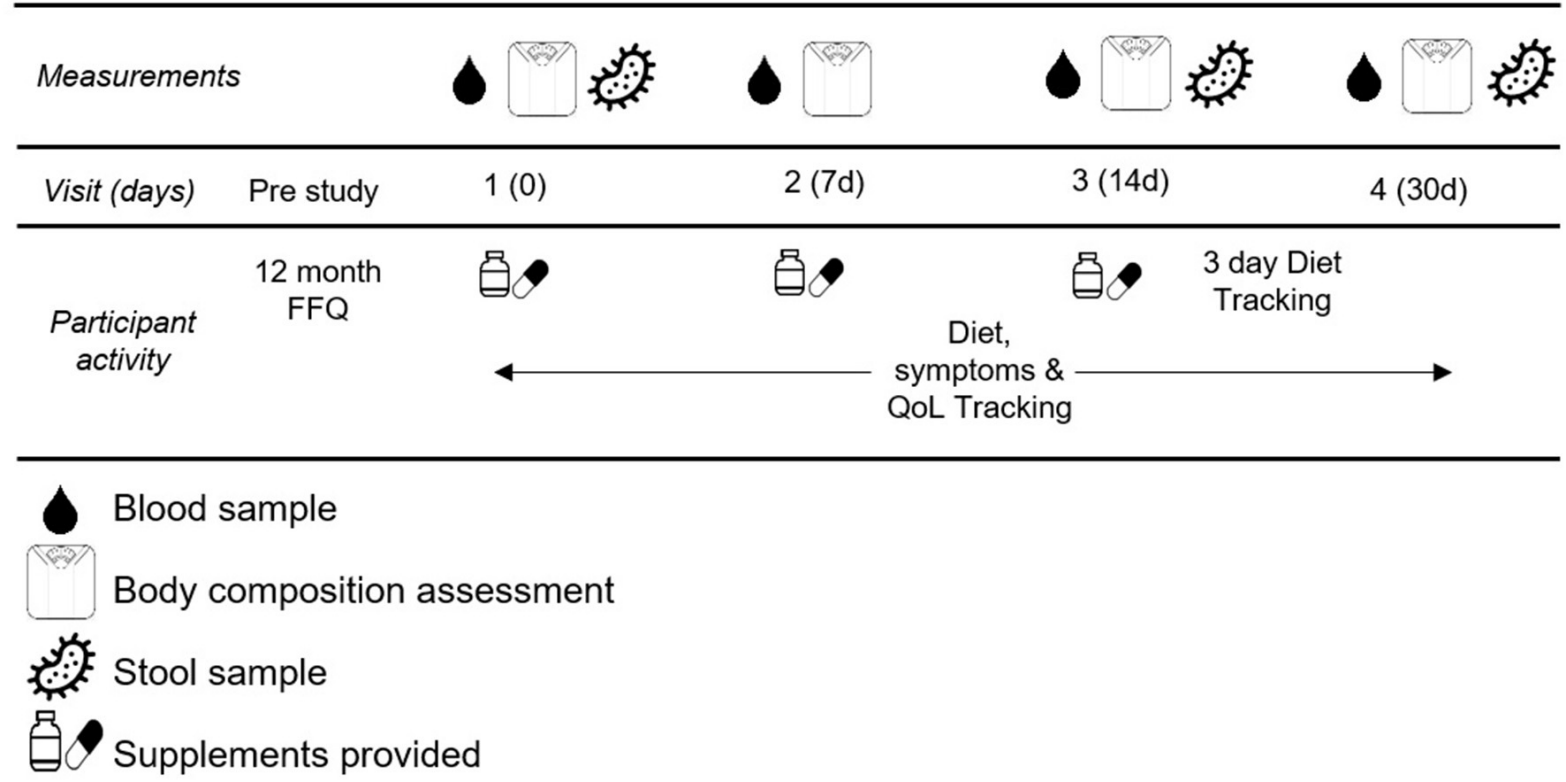
Testing Schematic.

This study was a double-blind randomized control study. Participants completed five total visits for this study. The first visit was to obtain informed consent and explain the procedures thoroughly. The remaining four visits were scheduled for blood draw, blood pressure assessment (Omron HEM-711), body composition assessment (Bioelectrical impedance, InBody USA), and fecal sample collection. These occurred in the morning after a 10 h fast at baseline, and after 7, 14, and 30 days of supplementation. See Figure 2 for testing schematic.

### Supplementation Protocol

MTC juice was prepared by diluting 1 fluid ounce of concentrate (King Orchards, Traverse City, MI) with 7 fluid ounces of filtered water in accordance with manufacturer instructions. The placebo was a visually similar carbohydrate- and calorie-matched placebo beverage. Participants were provided with 14, 240 ml (8 oz.) bottles of juice per week for 4 weeks. They were instructed to keep the juice refrigerated until consumption, to shake well at consumption, and to drink two bottles per day, ~8 h apart and not within an hour of exercise. Each bottle of MTC juice contained 77 kcal, 18 g carbohydrate and 0 g fiber and the equivalent of 0.5 lb of MTC per serving, while each bottle of placebo juice contained 67 kcal, 17 g carbohydrate and 0 g fiber. MTC capsules (King Orchards, Traverse City, MI) contained 500 mg of freeze-dried tart cherries, while placebo capsules contained 460 mg cornstarch. Participants were provided with an undisclosed number of capsules and instructed to take two capsules with breakfast each day and to return unused capsules at their next visit. Each MTC capsule contained 1.7 kcal, 0.4 g carbohydrate and 0.2 g fiber and contained 0.92 ounces of cherry skins and pulp. Each placebo capsule contained 1.8 kcal, 0.4 g carbohydrate and 0 g fiber. Independent lab analysis of anthocyanins *via* high-performance liquid chromatography and total polyphenols *via* Ultraviolet-Visible Spectroscopy (Certified Laboratories, Mellville, NY) showed MTC juice provided 227 mg anthocyanins and 793 mg total polyphenols per 240 ml bottle while MTC capsules provided 330 mg anthocyanins and 760 mg total polyphenols per capsule. These values are within the ranges reported by previous studies using MTC products (29). Doses were in accordance with previous literature and to ensure similar levels of polyphenols and anthocyanins between formulations. Two capsules were taken at the same time of day rather than spread out for easier compliance. Compliance for both juices was 100%, while it was 92% for placebo capsules and 94% for MTC capsules.

### Diet and Exercise Tracking

Following the consent visit and prior to their baseline blood draw, participants completed a 12 month food frequency questionnaire (FFQ) that included portion sizes (Dietary History Questionnaire (DHQ) III, National Cancer Institute) to determine typical dietary patterns (i.e., high, low, or normal intake of carbohydrate, protein, fat, and fiber based on established dietary recommendations). In addition to the FFQ, between the 14 and 30 day visits, participants completed a 3 day food record for 2 weekdays and 1 weekend day, tracking food and beverage intake along with portion sizes. Participants recorded their data in Food Prodigy, a companion program to the Food Processor Nutrition Analysis software (ESHA Research). Data from the 3 days was then exported from the Food Processor Nutrition Analysis program as an excel file for each participant. Finally, every 7 days during the course of the study participants completed an online survey regarding their exercise and dietary habits for the previous 7 days as well as the frequency of their intake of alcohol, anti-inflammatory medications, and the top 100 polyphenol containing foods.

### Gut Microbiome Analysis

Fecal samples were collected by participants into a toilet hat (Protocult 100, Ability Building Center, Minneapolis, MN) and aliquoted into three Polypropylene microcentrifuge tubes that were free from DNase/RNase and pyrogens (Eppendorf™ Biopur®). Samples were immediately frozen at −20°C until delivery to the laboratory where they were stored at −80°C. Samples were transported to the lab on ice (Utek, Sonoco ThermoSafe, Arlington Heights, IL) in a thermal insulated tote (Hopkins Medical Products Caledonia, MI).

DNA was extracted from fecal samples using the Qiagen DNeasy PowerSoil kit (Qiagen, 12888-100) per manufacturer's instructions. Isolated genomic DNA was amplified using custom designed primers targeting the 16s rDNA V3-V4 regions with Kapa Biosystems HiFi HotStart ReadyMix (Roche, KK2601). Amplified products were checked for the correct size using the Agilent 2100 Bioanalyzer on a DNA 1000 chip (Agilent, 5067-1504). Each amplified product was dual indexed using Illumina Nextera XT v2 indices (Illumina, FC-131-200X) according to manufacturer's instructions. Prepared libraries were checked for correct sizing and overall quality using the Agilent 2100 Bioanalyzer on a DNA 1000 chip (Agilent, 5067-1504). Libraries were quantified using a Qubit 3.0 Fluorometer (Invitrogen, Q32850) and pooled equimolarly. Prepared libraries were mixed with 10% PhiX and sequenced on an Illumina MiSeq using a 2 × 300 bp (600 cycle) paired-end sequencing kit (Illumina, MS-102-3003). Sequencing reads were downloaded from the BaseSpace server in FASTQ format. Reads were demultiplexed and adaptors removed. Operational Taxonomic Units (OTUs) were generated using QIIME 2.0 and identified against the SILVA database. Species richness and diversity were calculated with the Shannon index and Pielou's evenness (Supplementary Figure). The datasets presented in this study can be found at https://www.ncbi.nlm.nih.gov/bioproject/PRJNA742936.

### Blood Sample Analysis

Venous blood samples were collected from an antecubital vein into Ethylenediaminetetraacetic acid (EDTA) and serum separator tubes (SST) immediately before and after 7, 14, and 30 days of supplementation. One milliliter of EDTA blood was used to quantify erythrocyte sedimentation rate (ESR) by the Westegren method (Sedi-Rate, Globe Scientific, Inc., Mahwah, NJ). The remaining EDTA blood was stored at 4°C until centrifugation. SST tubes were stored at room temperature for 30 min then centrifuged with EDTA tubes for 15 min (2,500 × g) and 4°C after which supernatants were collected and stored at −80°C for later analysis. Two milliliter serum were sent to an outside facility (Pathology Laboratories, Inc., Toledo, OH) for measurement of uric acid (UA) and C-reactive protein (CRP). Remaining serum samples were assayed in house in duplicate for TNF-a (BMS223HS, Invitrogen, ThermoFisher Scientific, Vienna, Austria), insulin (Catalog #90095, Crystal Chem, Elk Grove, IL), glucose (Item #120003100P, Eton Bioscience, San Diego, CA), and glycated albumin (Catalog # IT3979, G-Biosciences, St. Louis MO). HOMA-IR was calculated as (insulin x glucose)/405 (30). Reference range for CRP was 0.00–0.744 mg·dL^−1^ and 3.5 and 7.2 mg·dL^−1^ for males and females for UA.

### Statistical Analysis

A priori power analyses for between and within study design to attain 95% power and an alpha of 0.5 were conducted. For microbiota changes, 12 participants per group were needed for an effect size of 1.32 (10, 11). For UA, CRP, and ESR 12 participants per group were needed for an effect size of 0.72 (22), and 0.72 (18, 23), and 0.63 (23), respectively. For glucose a sample size of 12 was needed for an effect size of 0.75 and for insulin a sample size of 16 was needed for an effect size of 0.58 (28). Statistical analysis was completed using Statistical Package for the Social Sciences (SPSS; SPSS 26.0, Chicago, IL, USA). Linear mixed models were used to examine the main effects of time, and treatment, and the interaction effect (time x treatment). For microbiome composition, data was analyzed overall for change in OTUs between groups at baseline, 14 and 30 days. To overcome interindividual variability, a secondary analysis was conducted where participants were divided into groups based on their baseline levels of high (≥10%; *n* = 23) or low (<10%; *n* = 35) levels of *Bacteroides* because they have been shown to be the primary genus impacted by *in vitro* tart cherry treatment (11). Different covariance structures were systematically fit to the data, and the one that minimized the Hurvich and Tsai's criterion was chosen for the final model. Where a significant F ratio was observed, *post-hoc* comparisons with LSD-adjusted *p*-values were used to identify which pairs of means were significantly different. Normality and homogeneity of variance of the residuals were checked using Q-Q plots, and scatter plots, respectively, and deemed plausible in each instance. Two-tailed statistical significance was accepted as *p* < 0.05. Data are represented as mean ± SD.

## Results

### Gut Microbiome

**Figure 3:**
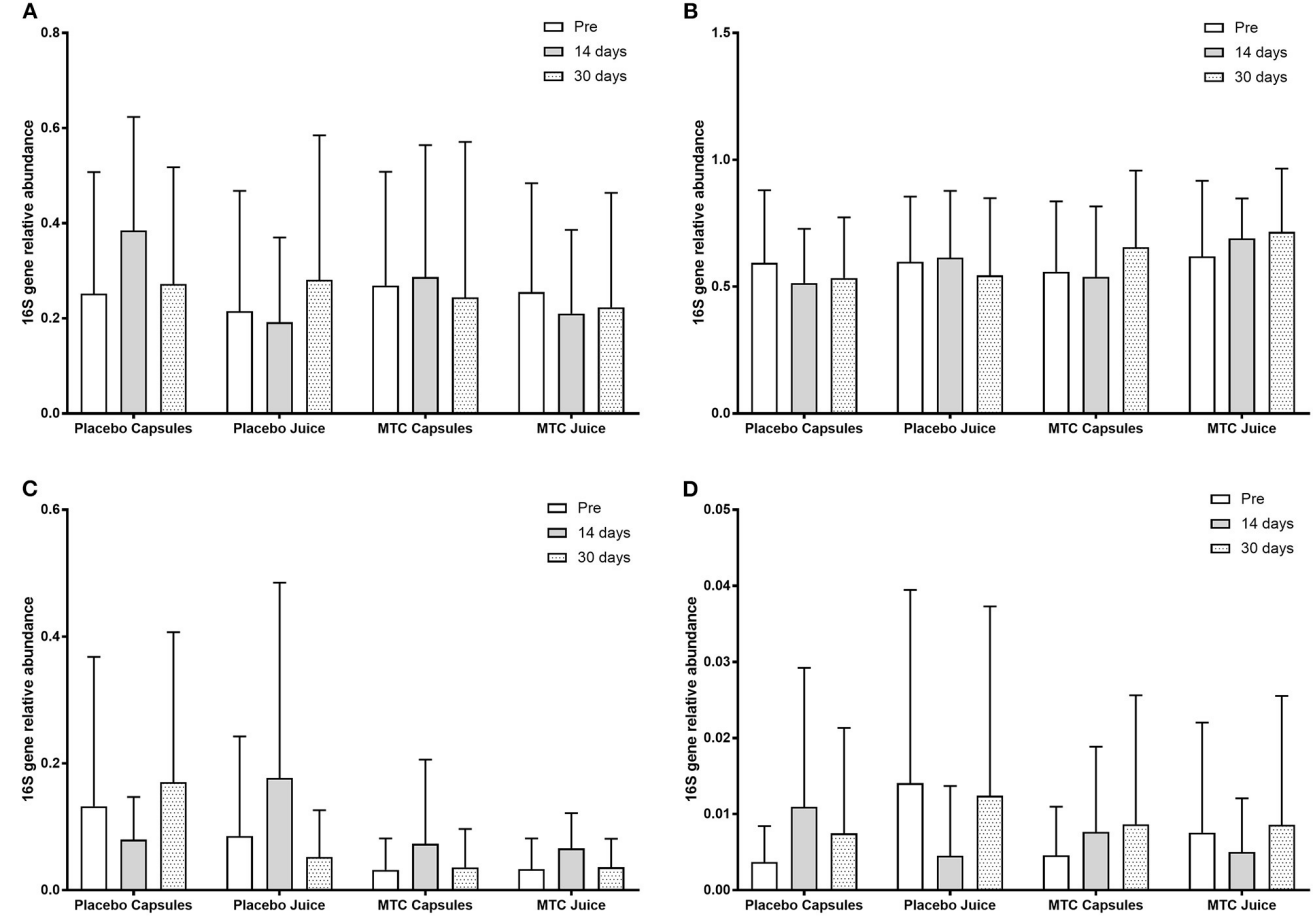
Observed taxonomic units (OTUs) for **(A)** Bacteroides, **(B)** Firmicutes, **(C)** Actinobacteria, and **(D)** Proteobacteria at baseline and after 14 and 30 days of supplementation with placebo or Montmorency tart cherry (MTC).

There was no significant change in bacterial OTUs over time (*F* = 0.70, *p* = 0.50) or between groups (*F* = 0.19, *p* = 0.91). When participants were divided by high or low levels of *Bacteroides* at baseline, there was a significant main effect for time (*F* = 4.10, *p* = 0.02) with greater *Bacteroides* at baseline vs. 14 days (*p* = 0.05) and 30 days (*p* = 0.01) and a significant interaction for time and *Bacteroides* level (*F* = 7.68, *p* = 0.001). However, for *Firmicutes* after dividing between high or low levels of *Bacteroides* there was no significant main effect for time (*F* = 0.60, *p* = 0.55) or group (*F* = 2.00, *p* = 0.16). Finally, there were no significant changes in any bacteria phyla or species over time or between groups. OTU data can be seen in Figure 3.

Correlations between the gut microbiome and other variables were mostly weak. BMI was positively correlated with *Lactobacillus* OTUs (*r* = 0.21, *p* = 0.01), *Bifidobacterium* OTUs (*r* = 0.21, *p* = 0.01), relative abundance of *Actinobacteria* (*r* = 0.23, *p* < 0.01), and relative abundance of *Bacteroidetes* (*r* = −0.25, *p* < 0.01). Glucose levels were correlated with relative abundances of *Bacteroidetes, Firmicutes, Proteobacteria* (*r* = 0.16, *p* = 0.04 for all), and *Faecalibacterium* (*r* = 0.23, *p* < 0.01). *Roseburia* OTUs were correlated with CRP (*r* = 0.19, *p* = 0.02).

### Inflammation

**Figure 4:**
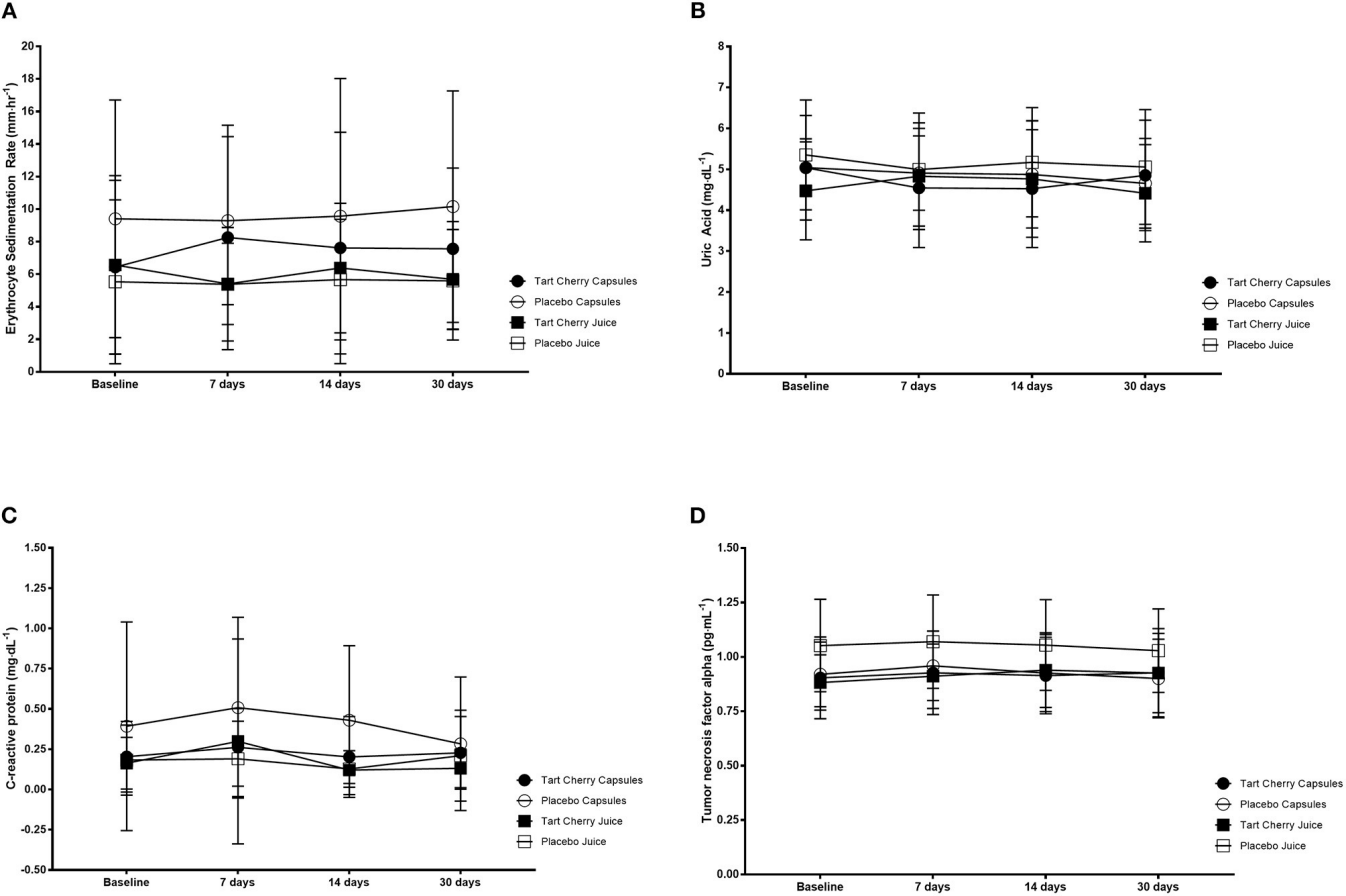
Inflammatory markers including erythrocyte sedimentation rate **(A)**, uric acid **(B)**, C-reactive protein **(C)** and tumor necrosis factor alpha **(D)** for each group at baseline and after 14 and 30 days of supplementation with placebo or Montmorency tart cherry (MTC) concentrate or capsules.

There was no significant change in ESR over time (*F* = 0.59, *p* = 0.63) or between groups (*F* = 1.82, *p* = 0.15). Similarly, there was no significant change in CRP over time (*F* = 0.94, *p* = 0.42) or between groups (*F* = 1.64, *p* = 0.19) nor did UA change over time (*F* = 1.26, *p* = 0.29) or between groups (*F* = 0.18, *p* = 0.91). For TNF-alpha, there was a significant change over time (*F* = 2.77, *p* = 0.04) with higher TNF-alpha at 14 vs. 30 days (*p* = 0.013; mean difference: 0.14 pg·ml^−1^, 95% CI: 0.02–0.27 pg·ml^−1^). However, there was no difference between groups (*F* = 2.37, *p* = 0.08). Inflammatory marker data can be seen in Figure 4.

### Blood Glucose Regulation

**Figure 5:**
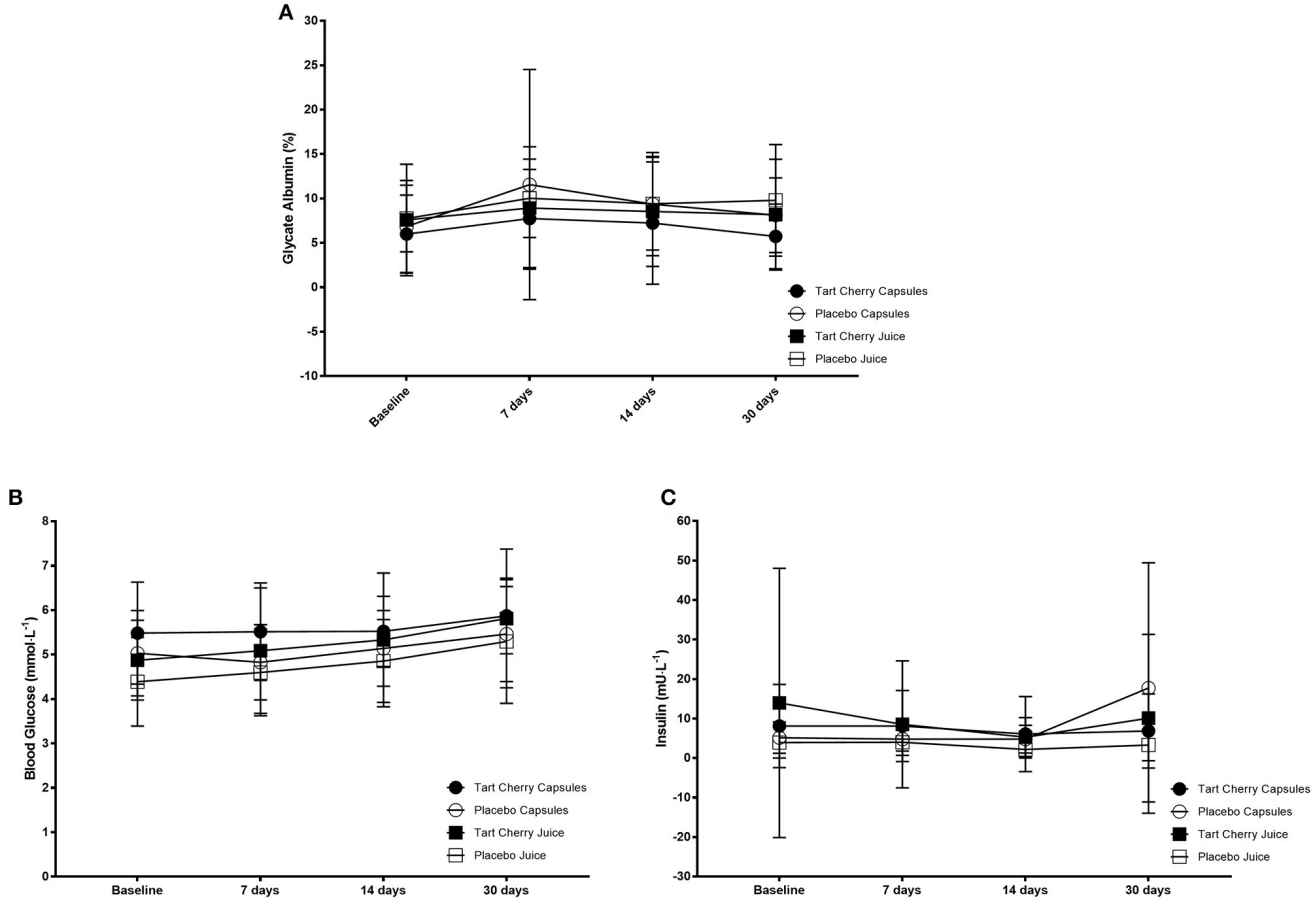
Glucose regulation markers including glycated albumin **(A)**, blood glucose **(B)**, and insulin **(C)** concentrations for each group at baseline and after 14 and 30 days of supplementation with placebo or Montmorency tart cherry (MTC) concentrate or capsules.

Fasting blood glucose levels steadily rose over the course of the study (*F* = 9.70, *p* < 0.001), with greater glucose at 14 days vs. baseline (*p* = 0.02; mean difference: 5.03 mmol·L^−1^, 95% CI: 0.75–9.30 mmol·L^−1^) and 7 days (*p* = 0.02; mean difference: 4.16 mmol·L^−1^, 95% CI: 0.52–7.80 mmol·L^−1^). Similarly, 30 days post was significantly higher vs. baseline (mean difference: 12.20 mmol·L^−1^, 95% CI: 7.18–17.21 mmol·L^−1^), 7 days (mean difference: 11.32 mmol·L^−1^, 95% CI: 6.89–15.76 mmol·L^−1^), and 14 days (mean difference: 7.17 mmol·L^−1^, 95% CI: 3.38–10.89 mmol·L^−1^) (*p* < 0.01 for all). However, there was no difference between groups (*F* = 1.17, *p* = 0.33). Insulin did not change over time (*F* = 1.79, *p* = 0.16), nor was it different between groups (*F* = 1.45, *p* = 0.24). Similarly, glycated albumin was not different over time (*F* = 2.39, *p* = 0.08) or between groups (*F* = 0.71, *p* = 0.56). Finally, HOMA-IR was not different over time (*F* = 1.07, *p* = 0.37) or between groups (*F* = 0.58, *p* = 0.63). Glucose regulation data can be seen in Figure 5.

### Dietary Intake

Completed FFQ and 3 day diet logs were compiled for 51/58 participants. Typical dietary pattern analysis found that all participants were classified as normal for protein intake, 39 participants were classified as normal, 4 classified as high, and 8 classified as low for carbohydrate intake. For fat, 36 were classified as normal and 15 were classified as high. For fiber, 16 participants were classified as normal, 11 were classified as high, and 24 were classified as low. There was no significant difference between groups in any of the macronutrients analyzed from the 3 day food log in this study (Table 2). Consumption of the top 100 polyphenol containing foods were tracked weekly, results can be found in Supplemental data. Total polyphenol consumption was 5,024 ± 4,599 for the MTC juice group, 4,744 ± 2,387 for the MTC capsule group, 2,519 ± 2,471 for the juice placebo group, and 3,424 ± 3,278 for the capsule placebo group and this was not significant between MTC formulations (*X*^2^ = 5.43, *p* = 0.14), but was higher for MTC vs. placebo group (*U* = 193, *p* = 0.02).

**Table 2:** Mean daily energy, alcohol, and nutrient intake of participants (*n* = 51) in the 3-day food log.

**Component (unit)**	**Mean**	**95% confidence interval**	**Between groups comparison (*p-*value)**	**Eta-squared**
Energy (kcal)	1,967	1,803–2,131	0.13	0.104
Carbohydrate (g)	246	221–271	0.08	0.125
Protein (g)	93	74–111	0.76	0.023
Total fat (g)	70	62–78	0.33	0.064
Dietary fiber (g)	26	21–31	0.70	0.027
Cholesterol (mg)	247	195–298	0.54	0.041
Total sugar (g)	78	67–88	0.26	0.076
Added sugar (g)	9	6–12	0.52	0.043
Caffeine (mg)	81	47–114	0.05	0.143
Average daily grain servings (oz)	6	5–7	0.37	0.059
Average daily vegetable servings (cups)	2	1–3	0.73	0.025
Average daily fruit servings (cups)	2	1–2	0.70	0.027

## Discussion

The current study examined the effects of 30 days of supplementing with MTC concentrate or freeze-dried powder on gut microbiome composition, inflammation, and glucose regulation. We hypothesized that the polyphenols in tart cherry products would influence the gut microbiome composition, which would modulate changes in inflammatory markers and glucose regulation. However, we found no significant alterations in the gut microbiome, and no significant impact of MTC supplementation on inflammation or glucose regulation.

### Gut Microbiome

The relationship between polyphenols and the gut microbiome has been established, whereby the degradation of most polyphenols requires host microbes and these microbes in turn utilize the products produced from polyphenol degradation for energy. This degradation of polyphenols often leads to greater bioavailability and biological activity (31) however this is dependent on the host microbiota composition (32). Tart cherries are well-known to be high in polyphenols and two previous investigations have examined the impact of tart cherry supplementation on the composition of the gut microbiome with disparate findings. Mayta-Apaza (11) investigated ingestion of 8 oz. MTC juice for 5 days in 10 participants. When comparing pre-to post-intervention microbiota, very little change was detected. However, the authors determined the need to divide participants into groups based on the baseline relative abundance of *Bacteroides*, either high or low. In the high *Bacteroides* group, ingestion of tart cherry juice resulted in a sharp decline in *Bacteroides* and an increase in *Firmicutes* (such as *Ruminococcus, Clostridium, Streptococcus* and Lactobacillus), while the opposite was seen in the low *Bacteroides* group, with an increase in *Bacteroides* and decreases in *Firmicutes* (such as *Streptococcus* and *Lachnospiraceae*). These changes may have been due to the underlying diets of the participants, as those in the low Bacteroides group consumed more carbohydrates, sugars, and fibers and the high polyphenol intake from the tart cherry juice resulted in increases in Bacteroides to facilitate breakdown of these polyphenols. When we divided our participants into groups based on high or low baseline levels of *Bacteroides*, we noted significantly higher *Bacteroides* in the high vs. low group at baseline and over the 30 days and no change in Firmicute levels, in contrast to the finding of Mayta-Apaza. However, we did not find any significant differences or changes in the other bacteria phyla or species, even if the groups were divided by their baseline *Bacteroides* levels.

Contrary to the findings of Mayta-Apaza, Lear (25) found no significant impact of 4 weeks supplementing with tart cherry concentrate on gut microbial composition. These authors noted their samples contained very low abundance of *Lactobacillus* and *Bifidobacterium*, which should have been more abundant and found their collection and storage methods may have resulted in alterations in their abundances. Nevertheless, our results are very similar to theirs despite our samples having an average 4% relative abundance of *Bifidobacterium* and 1% for *Lactobacillus*, well within the ranges of the healthy lower limits for these taxa (33). Early data suggests that the microbiome can be altered with dietary intake strategies, such as exclusively plant or animal based diets (6). For example, 20 days of red wine consumption increased *Firmicutes* and *Bacteroidetes* (8) and 30 days consumption of a high polyphenol cocoa drink increased bifidobacterial and lactobacilli populations. However, further analysis suggests these changes are transient, only lasting for 24 h after the consumption of high-fat/low fiber or low-fat/high fiber intake (34). In our study, it is possible that MTC supplementation brought change in microbial composition that might have not been measured with our fecal sample collection times (i.e., we missed the most opportune time for bacterial changes, although admittedly, we do not know what the most opportune timing for microbiome sampling following polyphenol consumption is since the majority of work is after very short (≤ 5 days) or long (>30 days) interventions). Further, Leeming et al. (35) note that the gut microbiota typically reverts back to its baseline state post-intervention, suggesting the need for long term dietary intervention. Therefore, future research should focus on detecting the sufficient time that bring the effective change in microbial composition from the intake of tart cherry supplementation.

It is also interesting to note that dietary intake of our participants did not seem to be a determining factor in whether or not the MTC supplementation altered microbial diversity, in contrast to Mayta-Apaza. Nearly 25% of our sample were considered to have high fat diets, while 40% had low fiber intake. While this likely determined whether a participant had high or low levels of *Bacteroidetes* at baseline, either the supplements did not provide enough of a stimulus for change, or the participant's dietary patterns were too influential to bring about change. This might indicate a need for greater changes in diet, along with MTC supplementation, to see significant alterations in gut microbiome composition.

### Inflammatory Markers

#### Erythrocyte Sedimentation Rate

When inflammation is present in the body, red blood cells stick to one another and this results in greater sedimentation rates. While this is a non-specific marker of inflammation, it has been measured in two previous studies using tart cherry juice consumption (23, 36). While a 4 week intervention found a modest decline in ESR of 5% (23), another 4 week intervention actually found a 17% increase in ESR (36). Our findings are similar to the latter, as interestingly we found an average 11% increase in ESR over the course of the study, and we did not find a significant difference in ESR between groups. The average values for ESR were well within the normal ranges for the test, indicating there was no measurable inflammation in our participants, which differs from the two previous studies. However, some time points were a bit higher, causing larger intra-individual variability (average CoV: 27%), potentially due to exercise and acute inflammation and suggest this might not be a marker to measure changes in inflammation with tart cherry supplementation in the future in healthy populations.

#### Uric Acid

Uric acid is produced in the body from the breakdown of purines, and if not metabolized itself, can accumulate resulting in pain and inflammation. There is significant interest in the use of tart cherries to reduce UA and the incidence of gout because much of the research indicates MTC concentrate (18), MTC juice (22, 23) and freeze-dried MTC decrease UA (22). The majority of studies investigating UA and tart cherry are acute in nature, with short supplementation periods (up to 48 h) with decreases of up to 36% during the initial 8 h after consumption (18, 22). Only one of the two longer-term studies (36) found a decrease (19%) in plasma urate while the other found a modest (2%) decline in UA after 120 days of supplementation with MTC concentrate (37). In the current study we also found a modest 2% decline in UA from baseline to 30 days, however there was no significant difference in UA between groups, which is surprising given the literature. Similar to ESR, the majority of the values for our participants were within the normal range, however there was large intra-individual variability (average CoV: 30%), which likely played a role in the lack of significant findings.

#### C-Reactive Protein

CRP is used as a marker for inflammation, typically used to predict cardiovascular disease risk. However, because it is an essential marker of inflammation and elevated CRP has been noted in many inflammatory diseases (38) it can be a valuable tool for evaluating the impact of tart cherry on inflammation. Equivocal findings have been noted with tart cherry supplementation and changes in CRP, potentially due to different lengths of supplementation and doses. Acute interventions with MTC juice or concentrate are equivocal. For example, Bell et al. (18) found a 29% decrease in CRP 5 h after ingestion of 30 or 60 ml MTC concentration, with no difference between the doses, and this reduction was still apparent at 48 h. However, Hillman and Uhranowsky (22) did not find a change in CRP during a 48 h intervention with either 30 or 60 ml MTC juice. Moreover, a 4 week intervention with MTC juice found a non-significant 19% reduction in CRP (23), while other interventions lasting 6 weeks (39) and 12 weeks (36, 40) also failed to find significant reductions in CRP from MTC supplementation. Two investigations that utilized sweet Bing cherries did find significant reductions in CRP both after 3 h (41) and 28 day (42). These different results could be due to formulations, as sweet tart cherries are the only ones known to have anthocyanins in all portions of the fruit (skin, flesh and pit), while tart cherries do not have anthocyanins in their flesh and very little in their pits, though tart cherries have higher antioxidant properties (43). Another reason for differences between studies could be due to type of CRP measured (inflammatory vs. high-sensitivity) or its reported low reproducibility (44). Levels of CRP in our study are similar to other investigations who used healthy participants and MTC supplements and perhaps the lack of change in our study is due to this healthier population used. Indeed studies utilizing participants with high baseline levels of CRP tend to find significant reductions supplementing 4–6 weeks with MTC supplementation (21, 36).

#### TNF Alpha

TNF-α is a pro-inflammatory cytokine that regulates many body processes including inflammatory reactions by stimulating additional pro-inflammatory cells. TNF-α is not typically present in healthy humans, but increased levels are often found in inflammatory bowel diseases, where it leads to pathological inflammation (45). TNF-α in humans has been decreased (36, 42) or unaffected by cherry ingestion (41, 46, 47), and animal models demonstrate similar results (48, 49). The current study actually found a modest increase (2%) in TNF-α over the course of the study, primarily driven by an increase from baseline to 7 days, with large increases in a few of the participants (10–21% increase in 10/58 participants). TNF-α is positively associated with relative abundance of bacteria such as *Ruminococcaceae, Blautia*, and *Lactobacillus* (45), however because we did not see a significant increase in these populations, we cannot speculate that this was the cause. One study has found elevated TNF-α following ingestion of a Jerte Valley cherry-based product, which they speculated was caused by elevations in melatonin content (50), however because we did not measure melatonin content, we cannot be sure what caused TNF-α to increase in our study.

Taken together, the results of this study indicate MTC supplementation in either concentrate or freeze-dried powder has little impact on inflammation in apparently healthy participants, which may in part be due to a lack of sufficient inflammation in participants to observe any effect of the supplements.

### Glucose Regulation

Cell-line work demonstrates MTC extract treatment leads to changes in key enzymes related to glucose regulation in diabetes, including alpha amylase (26). This would result in slower peak glucose levels and a longer time for carbohydrate digestion, which may decrease average blood glucose over time. Glucose regulation in the current study was assessed *via* measurement of glycated albumin, which is reflective of glycemia over a 2–3 week period (51), as well as blood glucose and insulin levels. Glycated albumin and insulin were not significantly changed over time nor were they different between groups, however blood glucose levels steadily rose over the course of the study but were not different between groups. This data is in line with the work by Lear (25) who found a decrease in insulin sensitivity (decreased Matsuda index), with an increase in insulin and no decrease in blood glucose in those supplementing with MTC juice. Additionally, Chai et al. (28) found an increase in blood glucose following 12 weeks of MTC concentrate ingestion, while Kimble et al. (52) found no impact of 12 weeks MTC concentrate ingestion on blood glucose levels. Interestingly, a seven day intervention in patients with metabolic syndrome found a significant decline in blood glucose and an increase in insulin (53), which suggests in the short-term, supplementation may improve insulin sensitivity, but this does not appear to translate to long-term benefits. Our findings show an elevation in glucose without a rise in insulin, which might indicate presence or development of insulin insensitivity, indeed 16 individuals (28%) had a fasting glucose >100 mg·dL^−1^ (but <126 mg·dL^−1^) at baseline and an additional 5 participants had elevated glucose levels from 7 days onward. Finally, the difference in sugar composition between the juice and capsule groups were significantly different (17.7 vs. 0.4 g), however there was no significant difference between groups in blood glucose regulation, it appears unlikely the results are due to greater sugar intake from the supplements and further human intervention work with MTC supplements and blood glucose regulation are needed.

## Conclusion

The current study hypothesized that the polyphenols in MTC products would influence the gut microbiome composition, which would modulate changes in inflammatory markers and glucose regulation. However, we found no significant alterations in the gut microbiome, and no significant impact of MTC supplementation on inflammation or glucose regulation. These results may partially be due to the use of a healthy population, who did not have inflammatory conditions and thus future work may need to focus on clinical populations. Additionally, time point measurements for the gut microbiome may have missed changes in bacterial composition, therefore additional time course analysis with more frequent measurements may be necessary to see if MTC has any impact on gut microbial composition.

## Data Availability Statement

The datasets presented in this study can be found in online repositories. The names of the repository/repositories and accession number(s) can be found below: https://www.ncbi.nlm.nih.gov/bioproject/PRJNA742936.

## Ethics Statement

The studies involving human participants were reviewed and approved by Ohio University Institutional Review Board at Ohio University (IRB protocol# 18-F-13). The patients/participants provided their written informed consent to participate in this study.

## Author Contributions

AH and BC contributed to conception, design of the study, performed the statistical analysis, and wrote sections of the manuscript. AH organized the database and wrote the first draft of the manuscript. All authors contributed to manuscript revision, read, and approved the submitted version.

## Funding

This study was funded by a grant from The Cherry Marketing Institute.

## Conflict of Interest

The authors declare that the research was conducted in the absence of any commercial or financial relationships that could be construed as a potential conflict of interest.

## Publisher's Note

All claims expressed in this article are solely those of the authors and do not necessarily represent those of their affiliated organizations, or those of the publisher, the editors and the reviewers. Any product that may be evaluated in this article, or claim that may be made by its manufacturer, is not guaranteed or endorsed by the publisher.
