# Supplementary figures and images for: Thirty Days of Montmorency Tart Cherry Supplementation Has No Effect on Gut Microbiome Composition, Inflammation, or Glycemic Control in Healthy Adults
Source: Front Nutr. 2021 Sep 16;8:733057. doi: 10.3389/fnut.2021.733057 (PMC8481367; doi:10.3389/fnut.2021.733057)

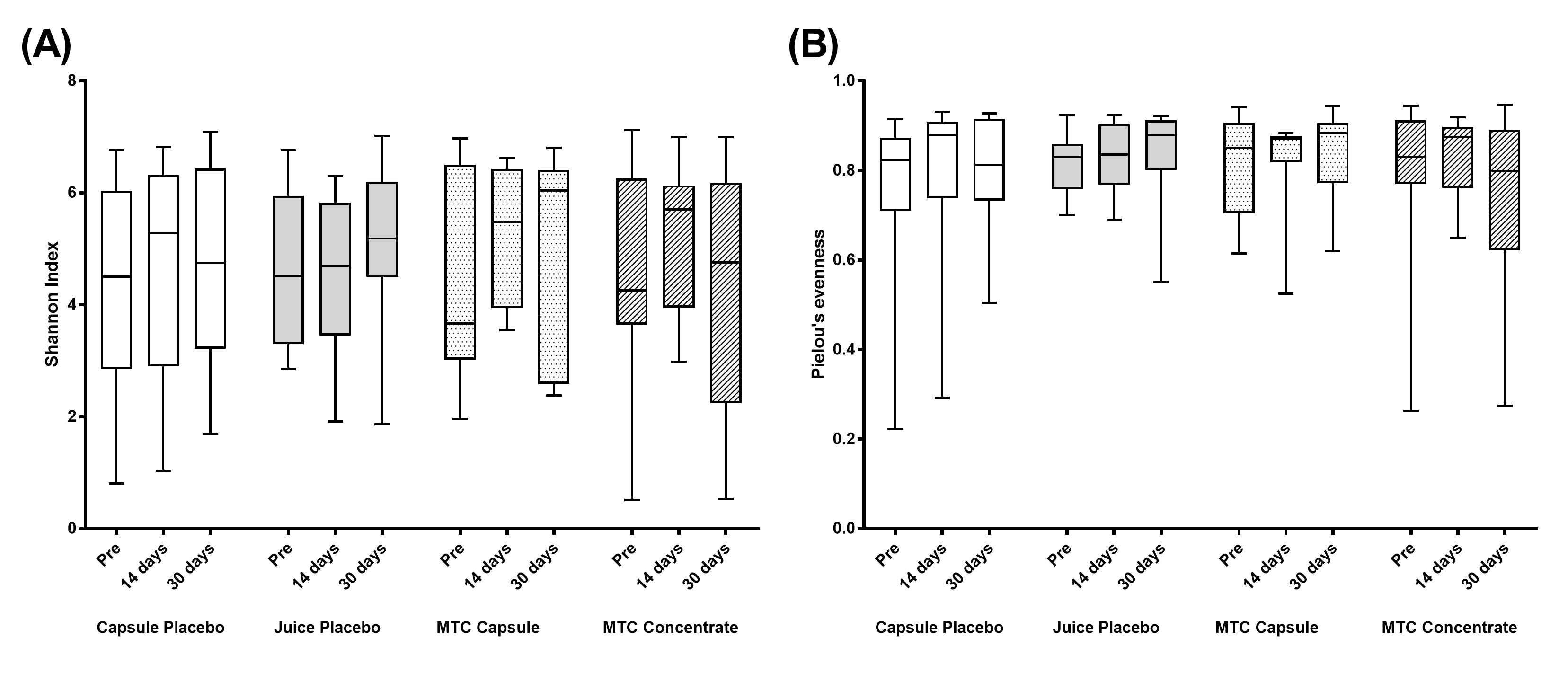

Supplement: Supplementary file 1 [file Image_1.TIF]
