# Supplementary material for: Thirty Days of Montmorency Tart Cherry Supplementation Has No Effect on Gut Microbiome Composition, Inflammation, or Glycemic Control in Healthy Adults
Source: Front Nutr. 2021 Sep 16;8:733057. doi: 10.3389/fnut.2021.733057 (PMC8481367; doi:10.3389/fnut.2021.733057)
Supplement: Supplementary file 2 [file Table_1.DOCX]

Supplementary Material

# Supplementary Data. Percentage of participants weekly consumption of top 100 polyphenol containing foods.

A. Spices

|  | Cloves | Cinnamon | Mint | Basil | Star anise | Rosemary | Sage | Oregano | Coriander | Turmeric | Nutmeg | Capers |
| --- | --- | --- | --- | --- | --- | --- | --- | --- | --- | --- | --- | --- |
| Never | 89% | 57% | 90% | 54% | 96% | 69% | 78% | 45% | 79% | 75% | 93% | 95% |
| 1-2 Times a Day | 0% | 7% | 1% | 1% | 0% | 0% | 0% | 0% | 0% | 0% | 0% | 0% |
| Once per week | 9% | 22% | 8% | 26% | 2% | 22% | 15% | 29% | 13% | 17% | 5% | 5% |
| 2-3 Times a Week | 2% | 13% | 3% | 18% | 2% | 9% | 7% | 21% | 9% | 7% | 3% | 2% |
| 4-6 Times a Week | 2% | 4% | 0% | 4% | 2% | 2% | 2% | 6% | 1% | 2% | 1% | 0% |
| 6+ times per week | 0% | 0% | 0% | 0% | 0% | 0% | 0% | 0% | 0% | 0% | 0% | 0% |

B. Fruit

|  | Blueberries | Grapes | Strawberries | Peaches | Apples |
| --- | --- | --- | --- | --- | --- |
| Never | 66% | 59% | 26% | 84% | 38% |
| 1-2 Times a Day | 0% | 5% | 3% | 1% | 2% |
| Once per week | 16% | 18% | 50% | 9% | 24% |
| 2-3 Times a Week | 16% | 15% | 19% | 5% | 29% |
| 4-6 Times a Week | 3% | 5% | 4% | 3% | 9% |

C. Vegetables

|  | Swiss chard | Spinach | Beetroot | Lettuce (red leaf, romaine, etc.) | Arugula | Asparagus | Carrot | Cabbage (also as sauerkraut) | Kale | Broccoli |
| --- | --- | --- | --- | --- | --- | --- | --- | --- | --- | --- |
| Never | 97% | 33% | 91% | 23% | 84% | 76% | 28% | 76% | 75% | 22% |
| 1-2 Times a Day | 0% | 5% | 0% | 5% | 1% | 0% | 7% | 0% | 1% | 3% |
| Once per week | 4% | 17% | 8% | 20% | 9% | 15% | 20% | 13% | 13% | 23% |
| 2-3 Times a Week | 1% | 29% | 2% | 40% | 6% | 9% | 35% | 11% | 9% | 40% |
| 4-6 Times a Week | 0% | 17% | 0% | 14% | 0% | 1% | 11% | 3% | 5% | 13% |
| 6+ times per week | 0% | 0% | 0% | 0% | 0% | 0% | 0% | 0% | 0% | 0% |

D. Drinks

|  | Coffee | Tea | Cider | Fruit juice |
| --- | --- | --- | --- | --- |
| Never | 34% | 51% | 95% | 65% |
| 1-2 Times a Day | 31% | 9% | 0% | 2% |
| 3-5 Times a Day | 8% | 3% | 0% | 0% |
| 6+ Times a Day | 0% | 0% | 0% | 2% |
| Once per week | 4% | 18% | 4% | 17% |
| 2-3 Times a Week | 12% | 15% | 2% | 14% |
| 4-6 Times a Week | 13% | 6% | 0% | 2% |

E. Seeds, nuts, and oils

|  | Chestnut | Walnut | Pistachio | Pecan | Hazelnut | Sesame seed | Sunflower | Peanuts | Flaxseed |
| --- | --- | --- | --- | --- | --- | --- | --- | --- | --- |
| Never | 97% | 78% | 95% | 77% | 92% | 69% | 75% | 31% | 77% |
| 1-2 Times a Day | 2% | 2% | 0% | 0% | 0% | 1% | 2% | 14% | 4% |
| Once per week | 3% | 11% | 6% | 18% | 7% | 15% | 11% | 20% | 9% |
| 2-3 Times a Week | 1% | 7% | 1% | 7% | 3% | 15% | 12% | 24% | 7% |
| 4-6 Times a Week | 0% | 4% | 1% | 0% | 0% | 2% | 2% | 11% | 5% |
| 6+ times per week | 0% | 0% | 0% | 0% | 0% | 0% | 0% | 0% | 0% |
